# Autophagy as an adaptive resistance mechanism to epigenetic therapy in cancer

**DOI:** 10.3389/fonc.2026.1985943

**Published:** 2026-10-05

**Authors:** Natalie L. Hakim, John Thomas Gialluisi, Sruthi Sureshkumar, Madison E. Gamble, Claudia M. Espitia, Kevin R. Kelly, Jennifer S. Carew, Steffan T. Nawrocki

**Affiliations:** 1 Division of Translational Medicine, Department of Medicine and University of Arizona Cancer Center, Tucson, AZ, United States; 2 Division of Hematology, University of Southern California Keck School of Medicine, Los Angeles, CA, United States

**Keywords:** autophagy, cancer, drug resistance, epigenetic regulation, tumor adaptation

## Abstract

Epigenetic therapies can reverse malignant transcriptional programs, but their activity is often constrained by adaptive stress responses that allow cancer cells to survive and recover. Autophagy is a central component of this adaptation. By sustaining metabolic homeostasis, mitochondrial fitness, proteostasis, and lysosomal recycling, autophagy can buffer the cellular injury produced by inhibitors of DNA methyltransferases, histone deacetylases, and bromodomain proteins. These observations provide a strong rationale for combining epigenetic therapies with autophagy inhibition. However, the field has too often equated autophagosome accumulation with increased autophagic flux and treatment-induced autophagy with a functional dependency. Moreover, the lysosomotropic agents used in early clinical studies incompletely inhibit autophagy and exert broader effects on lysosomal biology. Consequently, compelling preclinical activity has not translated into consistent clinical benefit. Here, we critically evaluate the mechanisms through which epigenetic therapies engage autophagy, distinguish correlative markers from causal evidence of resistance, and assess the preclinical and clinical data supporting combination strategies. We also define the major barriers to translation, including inadequate pharmacodynamic assays, a lack of predictive biomarkers, context-dependent effects on tumor and immune cells, and the limited potency and selectivity of current autophagy inhibitors. Defining when, where, and how autophagy becomes a targetable dependency will be essential to translate this compelling biology into an effective precision treatment.

## 1 Introduction

A fundamental component of cancer progression is the ability of malignant cells to adapt to metabolic and therapeutic stress (1). This adaptability is driven by cellular pathways that coordinate metabolic homeostasis and sustain survival under adverse conditions. Among these pathways, autophagy plays a central role in maintaining cellular fitness during stress. Autophagy is an evolutionarily conserved, lysosome-mediated degradation pathway that recycles intracellular components to support cellular homeostasis and metabolic adaptation (2, 3). The three main types of autophagy are macroautophagy, microautophagy, and chaperone-mediated autophagy (CMA), which vary in their mechanisms of cargo delivery to the lysosome (3). Macroautophagy (hereafter referred to as autophagy) is frequently dysregulated in cancer and has been implicated in tumor progression, metabolic adaptation, and therapeutic resistance. Autophagy begins with the activation of the ULK1 complex, which initiates autophagosome formation (4). Subsequently, activation of the Beclin-1-VPS34 complex promotes phagophore nucleation (5). Autophagosome formation requires conserved autophagy-related (ATG) proteins that coordinate membrane expansion and cargo sequestration (6). The autophagosome then fuses with the lysosome, where the sequestered cargo is degraded into reusable building blocks that are released back into the cytosol (6). Autophagic activity is regulated by nutrient and energy-sensing pathways. A central regulator of autophagy is mechanistic target of rapamycin complex 1 (mTORC1). Under nutrient-rich conditions, mTORC1 suppresses autophagy by phosphorylating and inhibiting the ULK1 complex. Alternatively, stress activates AMP-activated protein kinase (AMPK), which promotes autophagy by activating ULK1 (4). Given its central role in coordinating cellular responses to stress, autophagy is increasingly recognized as closely integrated with epigenetic mechanisms that regulate adaptive transcriptional programs in cancer. These two systems work together to influence how cells respond to metabolic stress, DNA damage, and therapeutic pressure (7).

Epigenetic regulation consists of reversible mechanisms that influence gene expression without altering the underlying DNA sequence, and these mechanisms are often disrupted in cancer. This dysregulation can alter gene expression, DNA repair, and stress responses, promoting malignant progression and resistance to treatment (8–10). The three predominant epigenetic mechanisms involved in cancer are DNA methylation, histone modifications, and RNA-mediated regulation. First, DNA methylation is a fundamental epigenetic modification that regulates gene expression through the addition of methyl groups to cytosine residues, most commonly at CpG dinucleotides. These methylation patterns are established and maintained by DNA methyltransferases (DNMTs), and their disruption is a prominent feature of cancer-associated epigenetic remodeling (11). Second, histone acetylation is regulated by histone acetyltransferases and histone deacetylases (HDACs), which modulate chromatin accessibility and transcriptional activity. In cancer, altered HDAC activity contributes to aberrant gene expression programs associated with tumor progression and therapeutic resistance (12). Finally, non-coding RNAs, such as microRNAs (miRNAs), also regulate gene expression at the post-transcriptional level by modulating messenger RNA (mRNA) stability and translation. In cancer, dysregulation of miRNA expression can alter oncogenic and tumor-suppressive signaling networks, contributing to tumor progression and therapeutic resistance (13). Together, these epigenetic mechanisms coordinate gene expression programs that influence cellular identity, stress adaptation, and therapeutic responses in cancer.

Although autophagy and epigenetic regulation have traditionally been studied as distinct processes, studies have shown that these pathways are functionally interconnected (14). Epigenetic mechanisms can regulate the transcription of autophagy-related genes (14, 15). Conversely, autophagy can influence epigenetic states by inducing DNMT3A-dependent DNA methylation (16). Metabolites such as acetyl-CoA and NAD^+^ can also regulate epigenetic enzyme activity (17). These metabolic changes can further influence chromatin regulation by altering the activity of histone acetyltransferases, HDACs, and histone methyltransferases, thereby shaping stress-responsive gene expression (18). These findings suggest that bidirectional crosstalk may help tumor cells coordinate metabolic adaptation with chromatin-based transcriptional programs during stress. Treatment-induced changes in autophagy markers should be distinguished from true changes in autophagic flux. Similarly, autophagy-mediated cytoprotection should be distinguished from durable persistence following treatment withdrawal. Here, we discuss the mechanisms underlying autophagy/epigenetic regulation crosstalk, identify critical knowledge gaps, and describe how co-targeting these pathways may improve therapeutic response.

## 2 Autophagy in cancer

### 2.1 Context-dependent roles of autophagy in cancer

Autophagy has context-dependent functions during cancer development, acting as a suppressor of malignant transformation in early stages, while supporting survival, metastasis, and therapy resistance in advanced tumors. Under normal physiological conditions, autophagy maintains cellular homeostasis by removing damaged organelles and protein aggregates, thereby limiting oxidative stress, genomic instability, and early tumorigenesis (19). Loss of autophagy-related genes such as *BECN1*, *ATG5*, or *ATG7* has been associated with increased accumulation of reactive oxygen species (ROS), DNA damage, and tumor-promoting cellular stress (19). However, the role of autophagy evolves as tumors develop. Once malignant transformation has occurred, cancer cells exploit autophagy to adapt to environmental and metabolic stress. This shift highlights the dual nature of autophagy in cancer, functioning as a tumor-suppressive mechanism during early disease, while supporting survival in advanced malignancies. In solid tumors, this survival role is shaped by the tumor microenvironment, where abnormal vascularization creates regions of hypoxia and nutrient deprivation (20). Although hematologic malignancies such as acute myeloid leukemia (AML) do not form poorly vascularized tumor masses, malignant cells can experience hypoxic stress within the bone marrow microenvironment. In AML, autophagy supports cell survival and therapeutic resistance by maintaining mitochondrial homeostasis during hypoxic and treatment-induced stress and contributing to the maintenance of leukemic stem cell populations (21). These findings illustrate a broader principle: the therapeutic relevance of autophagy depends less on its basal activity than on whether a specific tumor has become dependent on the pathway for survival.

### 2.2 Therapy-induced autophagy and current research gaps

Therapy-induced autophagy is an important mechanism of tumor cell adaptation. A wide variety of anticancer therapies, including chemotherapy, targeted agents, and radiotherapy, have been shown to induce autophagy in tumor cells (22). In many cancer contexts, therapy-induced autophagy functions as a cytoprotective response that allows cancer cells to tolerate treatment-induced stress and contributes to therapeutic resistance. Consequently, pharmacologic inhibition of autophagy has gained significant interest as a strategy to enhance the efficacy of anticancer therapies. However, previous autophagy inhibition strategies utilizing chloroquine (CQ) and hydroxychloroquine (HCQ) have not consistently produced durable antitumor responses despite investigation in over 30 clinical trials (23). Collectively, these trials demonstrated that HCQ/CQ could not completely inhibit autophagy at tolerable doses (24–26).

New agents to inhibit autophagy at both the proximal (ULK1/2; DCC-3116) and distal points (lysosome; GNS561) are under clinical investigation with additional novel agents in preclinical development (27–30). GNS561 produces lysosomal dysfunction and antitumor activity in hepatocellular carcinoma models, although the divergent consequences of acute and chronic palmitoyl-protein thioesterase 1 (PPT1) inhibition underscore the importance of treatment schedule and biological context. Similarly, ULK1/2 inhibition can enhance the activity of KRAS^G12C^-directed therapy in lung cancer models, whereas ULK1 performs context-dependent functions in ovarian cancer spheroids (27–30). These observations argue against treating autophagy inhibition as a uniform therapeutic strategy across tumor types.

In addition to the need for new and more potent autophagy inhibitors, another gap in the field is the limited number of biomarkers that may predict which patients will respond best to therapy. Previous clinical trials rarely integrated specific autophagy markers into the study design. Instead, they monitored autophagy through autophagosome accumulation, LC3B localization and expression, and the selective degradation of SQSTM1/p62 (25, 31–33). Recent work also identified *DDIT4*/REDD1 as a potential predictive biomarker and high expression promotes mTORC1 inhibition and subsequent triggering of autophagy (34). However, its predictive value remains disease-specific and requires clinical validation. Genetic alterations across the broader autophagy network may also create dependencies that cannot be captured by a single marker, as illustrated by recurrent disruption of the autophagy interactome in myeloid neoplasms (35). Single-cell and multi-omic profiling may help define these biological states, but such approaches must be paired with functional assays of autophagic flux and treatment response. The central challenge is therefore not simply to identify tumors with active autophagy, but to identify those in which autophagy represents a necessary and therapeutically actionable survival mechanism (**Figure 1**).

**Figure 1:**
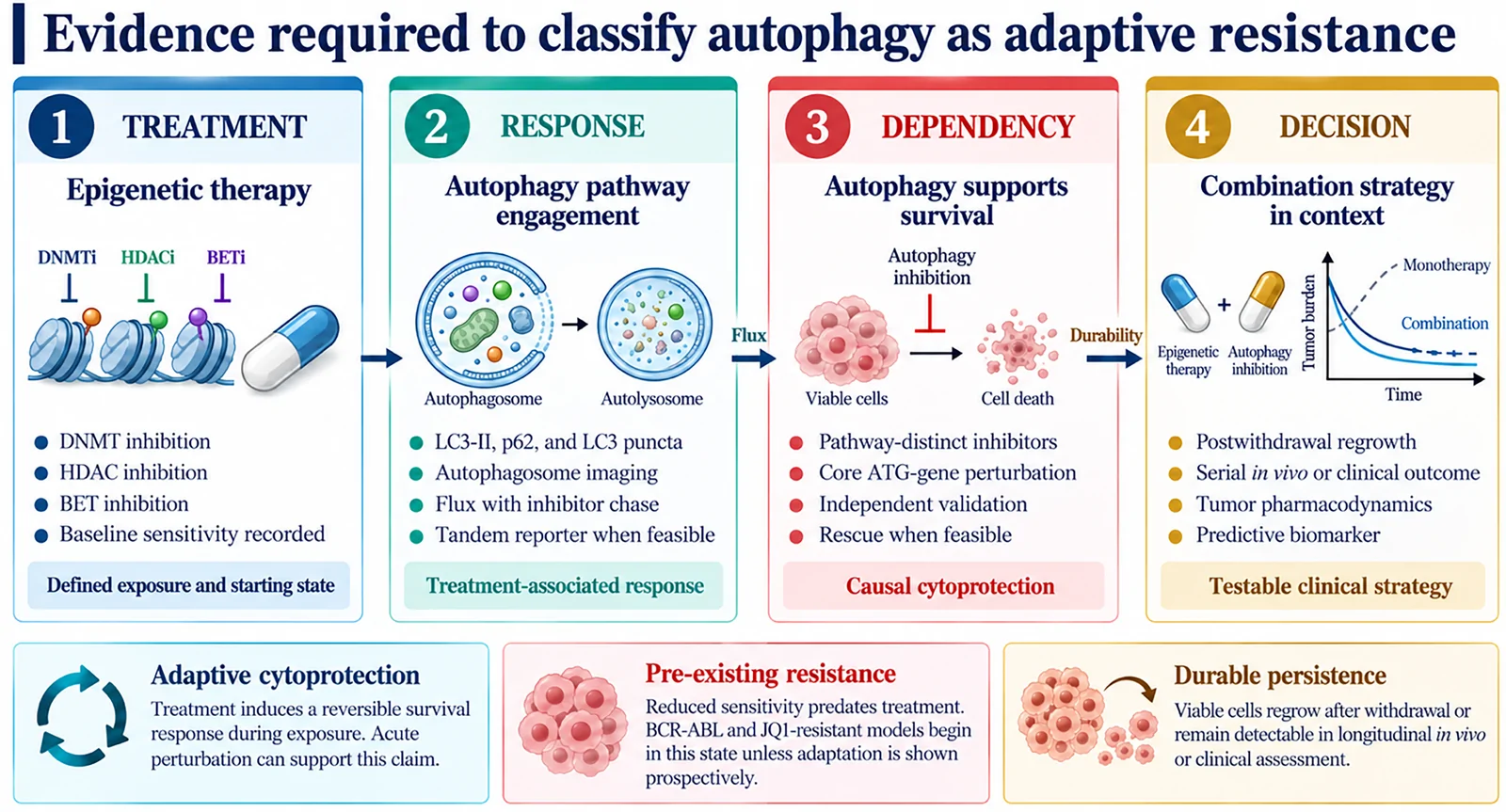
Evidence framework for assigning autophagy as an adaptive resistance mechanism to epigenetic therapy. Epigenetic treatment can change autophagy-associated markers, but pathway engagement alone does not establish increased flux or a survival dependency. Dynamic flux should be paired with orthogonal pharmacologic and genetic perturbation, with rescue where feasible. Claims of durable resistance require postwithdrawal regrowth, longitudinal *in vivo* evidence, or clinical outcomes linked to tumor pharmacodynamics. These thresholds distinguish treatment-induced cytoprotection from resistance that predates treatment and from durable persistence, and they define when an autophagy-directed combination is justified.

## 3 Epigenetic regulation in cancer

### 3.1 Epigenetic control of gene expression

Epigenetic dysregulation is a hallmark of cancer that drives transcriptional reprogramming through altered activity of epigenetic modifiers. These changes can disrupt tumor-suppressive programs, DNA repair, and stress-response pathways, promoting cancer cell plasticity and therapeutic resistance (36, 37). Promoter hypermethylation can silence genes involved in cell cycle control, apoptosis, and DNA repair, reducing mechanisms that normally limit uncontrolled proliferation and genomic instability (38, 39). Altered histone modification patterns can reshape chromatin accessibility in cancer, changing the expression of genes involved in proliferation, apoptosis, DNA repair, and therapeutic response (40). Non-coding RNAs, including miRNAs, regulate gene expression by affecting mRNA stability and translation (41). Together, these mechanisms show how epigenetic dysregulation can reshape transcriptional programs that control cancer cell behavior and treatment responses.

### 3.2 Cancer cell plasticity and therapeutic resistance

Epigenetic alterations also contribute to tumor heterogeneity, a major driver of disease progression and therapeutic resistance (42). Distinct epigenetic states within tumors generate transcriptional heterogeneity that enables subsets of cells to adapt to metabolic stress, evade immune surveillance, and resist therapy (43–45). Through these mechanisms, epigenetic dysregulation alters gene regulation in cancer (8, 46). Importantly, these adaptive responses can occur independently of permanent genetic alterations, permitting reversible therapy-resistant states (47). The central role of epigenetic regulation in transcriptional control and therapeutic resistance has established epigenetic pathways as attractive therapeutic targets in cancer (46). In the clinical setting, DNMT inhibitors and HDAC-targeting therapies have demonstrated efficacy in hematologic malignancies, including azacitidine (AZA) in high-risk myelodysplastic syndrome (MDS) and acute myeloid leukemia (AML), vorinostat in cutaneous T-cell lymphoma, and panobinostat in relapsed/refractory multiple myeloma (48–50). However, epigenetic therapies can also induce adaptive survival pathways, including cytoprotective autophagy, which may limit therapeutic efficacy and promote resistance (25, 51, 52). These findings support combination strategies that target both epigenetic regulation and autophagy-mediated stress adaptation. Nevertheless, a more comprehensive understanding of the reciprocal mechanisms linking chromatin regulation, autophagy signaling, and therapeutic resistance is needed to identify new therapeutic vulnerabilities.

## 4 Mechanistic crosstalk between autophagy and epigenetic pathways

### 4.1 Epigenetic regulation of autophagy-related genes

In cancer cells, epigenetic mechanisms can alter the expression of genes required for autophagosome formation, lysosomal function, and stress adaptation. Epigenetic modifications can regulate autophagy at the transcriptional and post-transcriptional levels, thereby altering autophagic activity in response to cellular stress (53, 54). Methylation-dependent regulation of autophagy genes has context-dependent, and sometimes opposing, effects on cancer phenotypes: hypermethylation-driven silencing of *MAP1LC3Av1* impairs autophagy and promotes gastric carcinogenesis, whereas hypomethylation of *BECN1* increases autophagy and drives tamoxifen resistance in breast cancer (55). Similarly, miRNAs can regulate autophagy by targeting core autophagy transcripts; for instance, miR-30a suppresses *BECN1* expression and thereby modulates autophagic activity (56). These mechanisms link epigenetic regulation to autophagy-mediated stress adaptation. In addition, transcriptional regulators such as TFEB promote the expression of autophagy and lysosomal genes during nutrient stress, while histone-modifying enzymes can further influence these transcriptional programs through changes in chromatin state (57). A key epigenetic mechanism regulating autophagy-related gene expression is histone acetylation. Increased histone acetylation promotes gene expression by creating a more accessible chromatin state; conversely, HDACs repress gene expression through chromatin condensation and transcriptional silencing (58). Through these effects on chromatin accessibility, HDAC activity may influence the expression of autophagy-related genes. Consistent with this relationship, pharmacologic HDAC inhibition has been shown to modulate autophagy in multiple cancer contexts, although this response may involve both transcriptional regulation and non-histone signaling mechanisms (51, 59).

### 4.2 Autophagy-dependent effects on epigenetic regulation

While epigenetic mechanisms can regulate autophagy-related gene expression, autophagy can, in turn, influence epigenetic regulation by altering the turnover of nuclear and chromatin-associated components. One mechanism involves nucleophagy, in which nuclear material, including nuclear proteins, is delivered to the lysosome for degradation. In this context, LC3 can promote lamin B1 degradation in response to oncogenic or genotoxic stress, linking autophagy to changes in nuclear architecture and chromatin organization (60). Because lamin B1 contributes to the maintenance of higher-order chromatin architecture and gene regulation, its turnover may alter transcriptional programs in cancer cells (61).

### 4.3 Metabolite-dependent regulation of autophagy and chromatin

Acetyl-CoA availability can influence histone acetylation and stress-responsive gene expression. In glucose-deprived glioma models, AMPK-dependent nuclear translocation of ACSS2 promoted local acetyl-CoA production and histone acetylation at promoters of TFEB-regulated lysosomal and autophagy genes, thereby supporting their transcription (62). This mechanism demonstrates metabolite-dependent chromatin regulation of autophagy but does not establish that autophagic recycling broadly sustains histone acetylation. One-carbon metabolism generates S-adenosylmethionine (SAM), the major methyl donor for DNA and histone methyltransferases (17). Although autophagy-derived substrates could theoretically affect one-carbon metabolism, direct evidence that autophagy alters SAM availability or methylation-dependent gene regulation in cancer remains limited. Whether autophagy influences methylation responses following DNMT inhibition remains an important question. NAD^+^ availability regulates the activity of sirtuins, a family of NAD^+^-dependent deacetylases involved in metabolic stress responses. SIRT1 regulates autophagy by deacetylating ATG5, ATG7, ATG8, and Beclin-1, thereby linking cellular metabolic state to the autophagy machinery (63, 64). These findings demonstrate metabolite-dependent regulation of autophagy but do not establish that autophagy alters SIRT1-dependent chromatin regulation. Whether this relationship influences chromatin organization or therapy-resistant transcriptional states in cancer remains unclear.

## 5 Therapeutic implications of co-targeting autophagy and epigenetic pathways

### 5.1 Epigenetic therapy-induced autophagy

The interaction between autophagy and epigenetic regulation is therapeutically relevant because both pathways help malignant cells adapt to treatment pressure. In some treatment settings, autophagy can function as a protective response to epigenetic therapy, and autophagy inhibition has been shown to enhance the activity of agents such as vorinostat and AZA (51, 52). By recycling damaged cellular components and preserving metabolic homeostasis, autophagy may act as a compensatory survival pathway that limits the efficacy of epigenetic therapy and contributes to therapeutic resistance (25, 65). This relationship has been observed across multiple classes of epigenetic agents. Among DNA methyltransferase inhibitors, studies combining AZA with autophagy inhibition have shown enhanced antileukemic activity, supporting the idea that autophagy may limit therapeutic responses in some contexts (52, 66). In these studies, inhibition of autophagy in combination with AZA increased cytotoxicity and enhanced innate immune and adaptive T-cell responses. Similarly, HDAC and DNMT inhibitors can reactivate tumor suppressor programs and promote apoptosis, but they may also induce autophagic responses that protect cancer cells from therapeutic stress (65, 67, 68). Together, these findings support the idea that autophagy activation can limit responses to epigenetic agents and provide a rationale for combination strategies to overcome therapy resistance.

### 5.2 Rationale for combination strategies

Because autophagy can function as an adaptive survival pathway, inhibiting autophagy can enhance the effects of epigenetic therapy in cancer cells (14). A clinical study combining the HDAC inhibitor vorinostat with HCQ provided initial support for the feasibility of this approach (25). However, this study also revealed key limitations of CQ derivatives, including incomplete autophagy inhibition and dose-limiting toxicity (25, 32). These challenges have shifted attention toward the development of more potent autophagy inhibitors that target either lysosomal function or upstream components of the pathway, such as ULK1, VPS34, and ATG4B (26). ATG4B is an attractive target for clinical autophagy inhibition as it is the only core ATG component with enzymatic cysteine protease activity. Dual-mechanism inhibitors that simultaneously target specific autophagy proteins (i.e., ATG4B) and disrupt lysosomal function may offer another opportunity to maximize cancer cell cytotoxicity.

Preclinical and early clinical studies have evaluated autophagy-modulating strategies across several classes of epigenetic therapy, including HDAC inhibitors, DNA methyltransferase inhibitors, and bromodomain and extra-terminal domain (BET) inhibitors (**Table 1**). Although non-coding RNAs also regulate autophagy-related gene expression, current combination strategies have been more extensively evaluated with pharmacologic epigenetic therapies (56). For example, autophagy inhibition has been shown to enhance the activity of both HDAC and DNMT inhibitors. BET inhibitor studies further suggest that autophagy modulation may be relevant beyond established DNMT and HDAC inhibition (69). Novel isoform-selective inhibitors may also minimize toxicity while enhancing tumor cell death. These findings support a therapeutic rationale in which autophagy inhibition is used to prevent an adaptive survival response to epigenetic therapy rather than serving as another cytotoxic agent.

**Table 1:** Evidence supporting autophagy-dependent cytoprotection during epigenetic therapy and limits of the adaptive-resistance claim.

Therapy, context, and reference	Autophagy and flux assessment	Evidence for autophagy-specific causality	Functional endpoint	Interpretation supported
Vorinostat (SAHA) BCR-ABL-positive CML (51)	**Flux evidence.** Treatment-associated autophagy was assessed with morphologic and static pathway measures. No lysosomal inhibitor-chase or tandem-reporter flux assay was used.	**Causality.** CQ and 3-MA gave concordant sensitization. Cathepsin D knockdown supported a downstream death mechanism, but no core ATG perturbation or rescue was performed.	**Endpoint.** Acute apoptosis and viability at 24–48 h; 8-day clonogenic regrowth after a 24-h exposure and washout; primary imatinib-refractory samples.	**Supports.** Treatment-associated cytoprotection and a postwithdrawal clonogenic effect in models with pre-existing BCR-ABL/imatinib resistance. **Does not show.** Acquired vorinostat resistance, relapse, or *in vivo* efficacy.
Vorinostat HCT8 and HT29 colon cancer (65)	**Flux evidence.** LC3-II, acidic-vesicle and lysosomal measures, and ultrastructure supported pathway engagement. No contemporary dynamic flux assay was used.	**Causality.** CQ and 3-MA plus ATG7 siRNA provided pharmacologic and genetic convergence. No rescue experiment was reported.	**Endpoint.** Acute apoptosis; 10-day clonogenic regrowth after a 24-h exposure; 21-day HCT8 xenograft growth and tumor apoptosis.	**Supports.** Cytoprotective autophagy during vorinostat exposure, post-treatment regrowth, and *in vivo* combination activity. **Limit.** CQ pleiotropy and no clinical tumor pharmacodynamics.
Vorinostat + HCQ Advanced solid tumors; phase I, n=27 (25)	**Flux evidence.** Serial PBMC autophagic vacuoles by TEM did not change significantly. Paired tumor biopsies in two patients showed LC3B, p62, and cathepsin D accumulation by IHC. These were surrogate or static pharmacodynamic measures, not dynamic tumor flux.	**Causality.** The nonrandomized combination study had no monotherapy control, genetic test, independent clinical validation, or rescue.	**Endpoint.** Safety and maximum tolerated dose; serial RECIST response. One durable partial response and prolonged stable disease in two patients were reported.	**Supports.** Clinical feasibility and limited pharmacodynamic sampling. **Does not show.** That autophagy inhibition caused clinical activity or identifies a predictive biomarker.
Panobinostat + CQ Triple-negative breast cancer (67)	**Flux evidence.** Autophagy-related protein and vesicle measures supported treatment response and CQ-associated blockade, but no tandem-reporter or formal inhibitor-chase flux comparison was reported.	**Causality.** CQ pharmacology only; no core ATG genetic intervention, independent autophagy-selective validation, or rescue.	**Endpoint.** Acute apoptosis and proteotoxic stress; xenograft tumor burden and host survival.	**Supports.** Preclinical combination activity. **Limit.** CQ off-target effects prevent a firm autophagy-specific resistance claim, and durability after withdrawal was not tested.
Panobinostat + CQ Ovarian cancer (68)	**Flux evidence.** Autophagy-related protein and vesicle readouts were measured; CQ blockade was inferred from static accumulation without a tandem-reporter flux assay.	**Causality.** CQ pharmacology only; no core ATG genetic intervention, independent inhibitor validation, or rescue.	**Endpoint.** Acute proliferation, apoptosis, DNA damage, and DNA-repair readouts in cell models.	**Supports.** Synergistic acute cytotoxicity. **Limit.** CQ-induced DNA damage and impaired repair may contribute independently of autophagy; no persistence, *in vivo*, or clinical endpoint.
Azacitidine + ROC-325 AML (52)	**Flux evidence.** Azacitidine-induced LC3B puncta and ROC-325 effects were assessed using autophagy, lysosomal, and ultrastructural measures. No tandem-reporter or inhibitor-chase flux study was reported.	**Causality.** A single lysosomal inhibitor was used in the combination study; no core ATG genetic intervention, independent inhibitor validation, or rescue.	**Endpoint.** Acute viability and apoptosis; survival in a disseminated AML xenograft model.	**Supports.** *In vivo* combination activity. **Limit.** Autophagy-specific dependency remains incomplete, and human exposure, tumor pharmacodynamics, and biomarker data are absent.
Azacitidine AML and immune recognition (66)	**Flux evidence.** LC3-I/II and autophagy-detection assays showed pathway activation after azacitidine, but dynamic flux was not directly resolved.	**Causality.** Spautin-1 and SAR405 gave concordant effects; external genetic and chemical datasets supported ERE regulation. No rescue confirmed autophagy-specific dependence.	**Endpoint.** Four-day cell growth and survival; ERE transcripts, dsRNA, MHC-I peptide presentation, and patient immune correlates.	**Supports.** Treatment-induced autophagy that buffers proteotoxic stress and constrains azacitidine immunogenicity. **Does not show.** Postwithdrawal persistence or clinical benefit.
BET inhibitor JQ1 AML stem-cell-like and primary CD34+CD38- blasts (69)	**Flux evidence.** Beclin-1 induction, LC3-II lipidation, autophagosome formation, and p62 loss gave concordant evidence of pathway activation. No lysosomal inhibitor-chase or tandem-reporter assay was reported.	**Causality.** Pharmacologic autophagy inhibition, BECN1 siRNA, compound C, and PRKAA/AMPK-alpha knockdown provided orthogonal perturbation. No rescue was reported.	**Endpoint.** Acute apoptosis in JQ1-resistant leukemia stem-cell-like lines and primary AML blasts.	**Supports.** Autophagy-dependent survival during JQ1 exposure in a pre-existing resistant state. **Does not show.** That JQ1 induced the resistant state or that blockade prevents durable persistence or relapse.

Evidence categories. Dynamic flux distinguishes increased autophagosome formation from impaired degradation using an inhibitor-chase or tandem reporter. Adaptive cytoprotection is a treatment-induced, reversible survival response during exposure. Pre-existing resistance is present before treatment. Durable persistence requires survival or regrowth after withdrawal, or longitudinal *in vivo* or clinical evidence. *No rescue* indicates that the study did not restore the phenotype after a causally targeted perturbation.

3-MA, 3-methyladenine; AML, acute myeloid leukemia; ATG, autophagy-related; BET, bromodomain and extraterminal; CML, chronic myeloid leukemia; CQ, chloroquine; DNMT, DNA methyltransferase; dsRNA, double-stranded RNA; ERE, endogenous retroelement; HCQ, hydroxychloroquine; IHC, immunohistochemistry; MHC-I, major histocompatibility complex class I; PBMC, peripheral blood mononuclear cell; RECIST, Response Evaluation Criteria in Solid Tumors; SAHA, suberoylanilide hydroxamic acid; TEM, transmission electron microscopy.

## 6 Discussion and future directions

The rationale for combining epigenetic therapy with autophagy inhibition is compelling, but the strategy has not yet been clinically validated. Current autophagy inhibitors lack the potency, selectivity, and tolerability required to achieve sustained pathway inhibition in tumors. Equally important, the cancers most likely to become dependent on autophagy during epigenetic therapy have not been defined. Developing more potent inhibitors will therefore be necessary, but it will not be sufficient. The central challenge is to distinguish autophagy induction from autophagic dependence. This distinction is particularly important because autophagy can suppress early tumorigenesis in some settings, while supporting the survival of established tumors exposed to metabolic or therapeutic stress. Increased LC3-II, altered SQSTM1/p62 abundance, lysosomal expansion, or induction of autophagy-related genes may indicate pathway engagement, but none of these measurements alone demonstrates increased autophagic flux or establishes that autophagy is required for tumor cell survival. Therapeutic studies must combine dynamic flux measurements with genetic or pharmacologic pathway disruption to determine whether autophagy is functioning as a causal resistance mechanism.

Candidate biomarkers, including LC3-II and SQSTM1/p62 turnover, lysosomal activity, ATG gene induction, REDD1 expression, TFEB activation, and treatment-induced chromatin changes, may help identify relevant biological states. However, none has been validated as a predictive biomarker for combined epigenetic and autophagy-directed therapy. Future studies should integrate paired tumor sampling, pharmacodynamic confirmation of sustained pathway inhibition, functional dependency testing, and single-cell profiling of malignant and immune compartments. These approaches will be necessary to determine whether autophagy inhibition enhances tumor cell killing without compromising beneficial antitumor immune responses. The success of this strategy will depend less on generating additional empiric combinations than on identifying the correct tumor, treatment context, biomarker, and autophagy inhibitor. Only then can autophagy be targeted as a defined adaptive dependency rather than as a broadly presumed mechanism of resistance.

## References

[1] Pavlova NN Thompson CB . The emerging hallmarks of cancer metabolism. Cell Metab. (2016) 23:27–47. doi: 10.1016/j.cmet.2015.12.006 26771115 PMC4715268

[2] Levy JMM Towers CG Thorburn A . Targeting autophagy in cancer. Nat Rev Cancer. (2017) 17:528–42. doi: 10.1038/nrc.2017.53 28751651 PMC5975367

[3] Glick D Barth S Macleod KF . Autophagy: cellular and molecular mechanisms. J Pathol. (2010) 221:3–12. doi: 10.1002/path.2697 20225336 PMC2990190

[4] Kim J Kundu M Viollet B Guan KL . AMPK and mTOR regulate autophagy through direct phosphorylation of Ulk1. Nat Cell Biol. (2011) 13:132–41. doi: 10.1038/ncb2152 21258367 PMC3987946

[5] Russell RC Tian Y Yuan H Park HW Chang YY Kim J . ULK1 induces autophagy by phosphorylating Beclin-1 and activating VPS34 lipid kinase. Nat Cell Biol. (2013) 15:741–50. doi: 10.1038/ncb2757 23685627 PMC3885611

[6] Feng Y He D Yao Z Klionsky DJ . The machinery of macroautophagy. Cell Res. (2014) 24:24–41. doi: 10.1038/cr.2013.168 PMC3879710 24366339

[7] Fullgrabe J Klionsky DJ Joseph B . The return of the nucleus: transcriptional and epigenetic control of autophagy. Nat Rev Mol Cell Biol. (2014) 15:65–74. doi: 10.1038/nrm3716 24326622

[8] Dawson MA Kouzarides T . Cancer epigenetics: from mechanism to therapy. Cell. (2012) 150:12–27. doi: 10.1016/j.cell.2012.06.013 22770212

[9] Yu X Zhao H Wang R Chen Y Ouyang X Li W . Cancer epigenetics: from laboratory studies and clinical trials to precision medicine. Cell Death Discov. (2024) 10:28. doi: 10.1038/s41420-024-01803-z 38225241 PMC10789753

[10] Jones PA Baylin SB . The fundamental role of epigenetic events in cancer. Nat Rev Genet. (2002) 3:415–28. doi: 10.1038/nrg816 12042769

[11] Edwards JR Yarychkivska O Boulard M Bestor TH . DNA methylation and DNA methyltransferases. Epigenet Chromatin. (2017) 10:23. doi: 10.1186/s13072-017-0130-8 28503201 PMC5422929

[12] West AC Johnstone RW . New and emerging HDAC inhibitors for cancer treatment. J Clin Invest. (2014) 124:30–9. doi: 10.1172/jci69738 24382387 PMC3871231

[13] Peng Y Croce CM . The role of MicroRNAs in human cancer. Signal Transduct Target Ther. (2016) 1:15004. doi: 10.1038/sigtrans.2015.4 29263891 PMC5661652

[14] Peixoto P Grandvallet C Feugeas JP Guittaut M Hervouet E . Epigenetic control of autophagy in cancer cells: a key process for cancer-related phenotypes. Cells. (2019) 8. doi: 10.3390/cells8121656 31861179 PMC6952790

[15] Lapierre LR Kumsta C Sandri M Ballabio A Hansen M . Transcriptional and epigenetic regulation of autophagy in aging. Autophagy. (2015) 11:867–80. doi: 10.1080/15548627.2015.1034410 25836756 PMC4502732

[16] Gonzalez-Rodriguez P Cheray M Fullgrabe J Salli M Engskog-Vlachos P Keane L . The DNA methyltransferase DNMT3A contributes to autophagy long-term memory. Autophagy. (2021) 17:1259–77. doi: 10.1080/15548627.2020.1816664 PMC8143216 32876528

[17] Kaelin WG Jr. McKnight SL . Influence of metabolism on epigenetics and disease. Cell. (2013) 153:56–69. doi: 10.1016/j.cell.2013.03.004 23540690 PMC3775362

[18] Bannister AJ Kouzarides T . Regulation of chromatin by histone modifications. Cell Res. (2011) 21:381–95. doi: 10.1038/cr.2011.22 21321607 PMC3193420

[19] White E . The role for autophagy in cancer. J Clin Invest. (2015) 125:42–6. doi: 10.1172/jci73941 25654549 PMC4382247

[20] Mah LY Ryan KM . Autophagy and cancer. Cold Spring Harb Perspect Biol. (2012) 4:a008821. doi: 10.1101/cshperspect.a008821 PMC3249624 22166310

[21] Dykstra KM Fay HRS Massey AC Yang N Johnson M Portwood S . Inhibiting autophagy targets human leukemic stem cells and hypoxic AML blasts by disrupting mitochondrial homeostasis. Blood Adv. (2021) 5:2087–100. doi: 10.1182/bloodadvances.2020002666 33877295 PMC8095145

[22] Amaravadi RK Kimmelman AC Debnath J . Targeting autophagy in cancer: recent advances and future directions. Cancer Discov. (2019) 9:1167–81. doi: 10.1158/2159-8290.cd-19-0292 31434711 PMC7306856

[23] Gewirtz DA . The challenge of developing autophagy inhibition as a therapeutic strategy. Cancer Res. (2016) 76:5610–4. doi: 10.1158/0008-5472.can-16-0722 27634767 PMC5050139

[24] Carew JS Nawrocki ST . Drain the lysosome: development of the novel orally available autophagy inhibitor ROC-325. Autophagy. (2017) 13:765–6. doi: 10.1080/15548627.2017.1280222 28118053 PMC5388230

[25] Mahalingam D Mita M Sarantopoulos J Wood L Amaravadi RK Davis LE . Combined autophagy and HDAC inhibition: a phase I safety, tolerability, pharmacokinetic, and pharmacodynamic analysis of hydroxychloroquine in combination with the HDAC inhibitor vorinostat in patients with advanced solid tumors. Autophagy. (2014) 10:1403–14. doi: 10.4161/auto.29231 PMC4203517 24991835

[26] Jones TM Espitia C Wang W Nawrocki ST Carew JS . Moving beyond hydroxychloroquine: the novel lysosomal autophagy inhibitor ROC-325 shows significant potential in preclinical studies. Cancer Commun (Lond). (2019) 39:72. doi: 10.1186/s40880-019-0418-0 31706349 PMC6842502

[27] Brun S Bestion E Raymond E Bassissi F Jilkova ZM Mezouar S . GNS561, a clinical-stage PPT1 inhibitor, is efficient against hepatocellular carcinoma via modulation of lysosomal functions. Autophagy. (2022) 18:678–94. doi: 10.1080/15548627.2021.1988357 PMC9037544 34740311

[28] Crissey MAS Versace A Bhardwaj M Jain V Liu S Singh A . Divergent effects of acute and chronic PPT1 inhibition in melanoma. Autophagy. (2024) 21:1–13. doi: 10.1080/15548627.2024.2403152 39265628 PMC11760279

[29] Ghazi PC O'Toole KT Srinivas Boggaram S Scherzer MT Silvis MR Zhang Y . Inhibition of ULK1/2 and KRAS(G12C) controls tumor growth in preclinical models of lung cancer. Elife. (2024) 13. doi: 10.7554/elife.96992.3 PMC11364435 39213022

[30] Webb JD Borrelli MJ Valdes YR Shepherd TG . Divergent role of ULK1 to balance mitochondrial homeostasis and bioenergetics in ovarian cancer spheroids. Cancers (Basel). (2026) 18. doi: 10.3390/cancers18111746 42279329 PMC13255712

[31] Rangwala R Chang YC Hu J Algazy KM Evans TL Fecher LA . Combined MTOR and autophagy inhibition: phase I trial of hydroxychloroquine and temsirolimus in patients with advanced solid tumors and melanoma. Autophagy. (2014) 10:1391–402. doi: 10.4161/auto.29119 PMC4203516 24991838

[32] Rosenfeld MR Ye X Supko JG Desideri S Grossman SA Brem S . A phase I/II trial of hydroxychloroquine in conjunction with radiation therapy and concurrent and adjuvant temozolomide in patients with newly diagnosed glioblastoma multiforme. Autophagy. (2014) 10:1359–68. doi: 10.4161/auto.28984 24991840 PMC4203513

[33] Vogl DT Stadtmauer EA Tan KS Heitjan DF Davis LE Pontiggia L . Combined autophagy and proteasome inhibition: a phase 1 trial of hydroxychloroquine and bortezomib in patients with relapsed/refractory myeloma. Autophagy. (2014) 10:1380–90. doi: 10.4161/auto.29264 PMC4203515 24991834

[34] Carew JS Espitia CM Sureshkumar S Carrera Espinoza MJ Gamble ME Wang W . REDD1 is a determinant of the sensitivity of renal cell carcinoma cells to autophagy inhibition that can be therapeutically exploited by targeting PIM activity. Cancer Lett. (2025) 613:217496. doi: 10.1016/j.canlet.2025.217496 39892703 PMC11832319

[35] Visconte V Przychodzen B Han Y Nawrocki ST Thota S Kelly KR . Complete mutational spectrum of the autophagy interactome: a novel class of tumor suppressor genes in myeloid neoplasms. Leukemia. (2017) 31:505–10. doi: 10.1038/leu.2016.295 PMC5844476 27773925

[36] Baylin SB Jones PA . Epigenetic determinants of cancer. Cold Spring Harb Perspect Biol. (2016) 8. doi: 10.1101/cshperspect.a019505 27194046 PMC5008069

[37] Hanahan D . Hallmarks of cancer: new dimensions. Cancer Discov. (2022) 12:31–46. doi: 10.1158/2159-8290.cd-21-1059 35022204

[38] Gopalakrishnan S Van Emburgh BO Robertson KD . DNA methylation in development and human disease. Mutat Res. (2008) 647:30–8. doi: 10.1016/j.mrfmmm.2008.08.006 18778722 PMC2647981

[39] Baylin SB . DNA methylation and gene silencing in cancer. Nat Clin Pract Oncol. (2005) 2:S4–11. doi: 10.1038/ncponc0354 16341240

[40] Liu R Wu J Guo H Yao W Li S Lu Y . Post-translational modifications of histones: mechanisms, biological functions, and therapeutic targets. MedComm (2020). (2023) 4:e292. doi: 10.1002/mco2.292 37220590 PMC10200003

[41] Calin GA Croce CM . MicroRNA signatures in human cancers. Nat Rev Cancer. (2006) 6:857–66. doi: 10.1038/nrc1997 17060945

[42] Guo M Peng Y Gao A Du C Herman JG . Epigenetic heterogeneity in cancer. biomark Res. (2019) 7:23. doi: 10.1186/s40364-019-0174-y 31695915 PMC6824025

[43] Pang L Zhou F Liu Y Ali H Khan F Heimberger AB . Epigenetic regulation of tumor immunity. J Clin Invest. (2024) 134. doi: 10.1172/jci178540 39133578 PMC11178542

[44] Marine JC Dawson SJ Dawson MA . Non-genetic mechanisms of therapeutic resistance in cancer. Nat Rev Cancer. (2020) 20:743–56. doi: 10.1038/s41568-020-00302-4 33033407

[45] Flavahan WA Gaskell E Bernstein BE . Epigenetic plasticity and the hallmarks of cancer. Science. (2017) 357. doi: 10.1126/science.aal2380 28729483 PMC5940341

[46] Jones PA Issa JP Baylin S . Targeting the cancer epigenome for therapy. Nat Rev Genet. (2016) 17:630–41. doi: 10.1038/nrg.2016.93 27629931

[47] Sharma SV Lee DY Li B Quinlan MP Takahashi F Maheswaran S . A chromatin-mediated reversible drug-tolerant state in cancer cell subpopulations. Cell. (2010) 141:69–80. doi: 10.1016/j.cell.2010.02.027 20371346 PMC2851638

[48] Fenaux P Mufti GJ Hellstrom-Lindberg E Santini V Finelli C Giagounidis A . Efficacy of azacitidine compared with that of conventional care regimens in the treatment of higher-risk myelodysplastic syndromes: a randomised, open-label, phase III study. Lancet Oncol. (2009) 10:223–32. doi: 10.1016/s1470-2045(09)70003-8 19230772 PMC4086808

[49] Olsen EA Kim YH Kuzel TM Pacheco TR Foss FM Parker S . Phase IIb multicenter trial of vorinostat in patients with persistent, progressive, or treatment refractory cutaneous T-cell lymphoma. J Clin Oncol. (2007) 25:3109–15. doi: 10.1200/jco.2006.10.2434 17577020

[50] San-Miguel JF Hungria VT Yoon SS Beksac M Dimopoulos MA Elghandour A . Panobinostat plus bortezomib and dexamethasone versus placebo plus bortezomib and dexamethasone in patients with relapsed or relapsed and refractory multiple myeloma: a multicentre, randomised, double-blind phase 3 trial. Lancet Oncol. (2014) 15:1195–206. doi: 10.1016/s1470-2045(14)70440-1 25242045

[51] Carew JS Nawrocki ST Kahue CN Zhang H Yang C Chung L . Targeting autophagy augments the anticancer activity of the histone deacetylase inhibitor SAHA to overcome Bcr-Abl-mediated drug resistance. Blood. (2007) 110:313–22. doi: 10.1182/blood-2006-10-050260 17363733 PMC1896119

[52] Nawrocki ST Han Y Visconte V Przychodzen B Espitia CM Phillips J . The novel autophagy inhibitor ROC-325 augments the antileukemic activity of azacitidine. Leukemia. (2019) 33:2971–4. doi: 10.1038/s41375-019-0529-2 31358855 PMC7462348

[53] Shin HR Kim H Kim KI Baek SH . Epigenetic and transcriptional regulation of autophagy. Autophagy. (2016) 12:2248–9. doi: 10.1080/15548627.2016.1214780 27487449 PMC5103355

[54] Ma Y Lv W Guo Y Yin T Bai Y Liu Z . Histone demethylases in autophagy and inflammation. Cell Commun Signal. (2025) 23:24. doi: 10.1186/s12964-024-02006-w 39806430 PMC11727796

[55] Shu F Xiao H Li QN Ren XS Liu ZG Hu BW . Epigenetic and post-translational modifications in autophagy: biological functions and therapeutic targets. Signal Transduct Target Ther. (2023) 8:32. doi: 10.1038/s41392-022-01300-8 36646695 PMC9842768

[56] Zhu H Wu H Liu X Li B Chen Y Ren X . Regulation of autophagy by a beclin 1-targeted microRNA, miR-30a, in cancer cells. Autophagy. (2009) 5:816–23. doi: 10.4161/auto.9064 19535919 PMC3669137

[57] Settembre C Di Malta C Polito VA Garcia Arencibia M Vetrini F Erdin S . TFEB links autophagy to lysosomal biogenesis. Science. (2011) 332:1429–33. doi: 10.1126/science.1204592 21617040 PMC3638014

[58] Kouzarides T . Chromatin modifications and their function. Cell. (2007) 128:693–705. doi: 10.1016/j.cell.2007.02.005 17320507

[59] Koeneke E Witt O Oehme I . HDAC family members intertwined in the regulation of autophagy: A druggable vulnerability in aggressive tumor entities. Cells. (2015) 4:135–68. doi: 10.3390/cells4020135 25915736 PMC4493453

[60] Dou Z Xu C Donahue G Shimi T Pan JA Zhu J . Autophagy mediates degradation of nuclear lamina. Nature. (2015) 527:105–9. doi: 10.1038/nature15548 26524528 PMC4824414

[61] Shah PP Donahue G Otte GL Capell BC Nelson DM Cao K . Lamin B1 depletion in senescent cells triggers large-scale changes in gene expression and the chromatin landscape. Genes Dev. (2013) 27:1787–99. doi: 10.1101/gad.223834.113 23934658 PMC3759695

[62] Li X Yu W Qian X Xia Y Zheng Y Lee JH . Nucleus-translocated ACSS2 promotes gene transcription for lysosomal biogenesis and autophagy. Mol Cell. (2017) 66:684–97:e9. doi: 10.1016/j.molcel.2017.04.026 28552616 PMC5521213

[63] Lee IH Cao L Mostoslavsky R Lombard DB Liu J Bruns NE . A role for the NAD-dependent deacetylase Sirt1 in the regulation of autophagy. Proc Natl Acad Sci USA. (2008) 105:3374–9. doi: 10.1073/pnas.0712145105 18296641 PMC2265142

[64] Sun T Li X Zhang P Chen WD Zhang HL Li DD . Acetylation of Beclin 1 inhibits autophagosome maturation and promotes tumour growth. Nat Commun. (2015) 6:7215. doi: 10.1038/ncomms8215 26008601 PMC4455096

[65] Carew JS Medina EC Esquivel JA Mahalingam D Swords R Kelly K . Autophagy inhibition enhances vorinostat-induced apoptosis via ubiquitinated protein accumulation. J Cell Mol Med. (2010) 14:2448–59. doi: 10.1111/j.1582-4934.2009.00832.x PMC2891399 19583815

[66] Noronha N Durette C Cahuzac M E Silva B Courtois J Humeau J . Autophagy degrades immunogenic endogenous retroelements induced by 5-azacytidine in acute myeloid leukemia. Leukemia. (2024) 38:1019–31. doi: 10.1038/s41375-024-02250-6 38627586 PMC11073987

[67] Rao R Balusu R Fiskus W Mudunuru U Venkannagari S Chauhan L . Combination of pan-histone deacetylase inhibitor and autophagy inhibitor exerts superior efficacy against triple-negative human breast cancer cells. Mol Cancer Ther. (2012) 11:973–83. doi: 10.1158/1535-7163.mct-11-0979 22367781

[68] Ovejero-Sanchez M Gonzalez-Sarmiento R Herrero AB . Synergistic effect of Chloroquine and Panobinostat in ovarian cancer through induction of DNA damage and inhibition of DNA repair. Neoplasia. (2021) 23:515–28. doi: 10.1016/j.neo.2021.04.003 33930758 PMC8100353

[69] Jang JE Eom JI Jeung HK Cheong JW Lee JY Kim JS . AMPK-ULK1-mediated autophagy confers resistance to BET inhibitor JQ1 in acute myeloid leukemia stem cells. Clin Cancer Res. (2017) 23:2781–94. doi: 10.1158/1078-0432.ccr-16-1903 27864418

